# Melatonin Suppressed the Heat Stress-Induced Damage in Wheat Seedlings by Modulating the Antioxidant Machinery

**DOI:** 10.3390/plants9070809

**Published:** 2020-06-28

**Authors:** Zeeshan Ali Buttar, Sheng Nan Wu, Marino B. Arnao, Chaojie Wang, Ikram Ullah, Chengshe Wang

**Affiliations:** 1 State Key Laboratory of Crop Stress Biology in Arid Areas and College of Agronomy, Northwest A&F University, Yangling 712100, Shaanxi, China; zeeshanbuttar@nwafu.edu.cn (Z.A.B.); wushengnan666@nwafu.edu.cn (S.N.W.); superjie_wang@nwafu.edu.cn (C.W.); 2 Department of Plant Biology (Plant Physiology), Faculty of Biology, University of Murcia, Campus de Espinardo, 30100 Murcia, Spain; marino@um.es; 3 College of Landscape and Horticulture, Yunnan Agriculture University, Kunming 650201, China; 2016071056@nwafu.edu.cn

**Keywords:** melatonin, wheat (*Triticum aestivum* L.), antioxidant enzymes, reactive oxygen species

## Abstract

Melatonin (*N*-acetyl-5-methoxytryptamine) is a pleiotropic signaling molecule that plays a crucial role in the regulation of various environmental stresses, including heat stress (HS). In this study, a 100 μM melatonin (MT) pretreatment followed by exposure to heat stress for different time periods was found to efficiently reduce oxidative stress by preventing the over-accumulation of hydrogen peroxide (H_2_O_2_), lowering the lipid peroxidation content (malondialdehyde (MDA) content), and increasing proline (Pro) biosynthesis. Moreover, the activities of antioxidant enzymes, such as superoxide dismutase (SOD), catalase (CAT), and peroxidase (POD), were increased substantially in MT-pretreated wheat seedlings. The presence of MT significantly improved the heat tolerance of wheat seedlings by modulating their antioxidant defense system, activating the ascorbate–glutathione (AsA–GSH) cycle comprising ascorbate peroxidase (APX), and increasing glutathione reductase (GR) activities. It also held the photosynthetic machinery stable by increasing the chlorophyll content. Enhancement in the endogenous MT contents was also observed in the MT+HS-treated plants. Furthermore, the expression of reactive oxygen species (ROS)-related genes TaSOD, TaPOD, and TaCAT, and anti-stress responsive genes, such as TaMYB80, TaWRKY26, and TaWRKY39, was also induced in MT-treated seedlings. Due to these notable changes, an improvement in stress resistance was observed in MT-treated seedlings compared with control. Taken together, our findings suggest that MT can play a key role in boosting the stress tolerance of plants by modulating the antioxidant defense system and regulating the transcription of stress-responsive genes.

## 1. Introduction

Wheat (*Triticum aestivum* L.) is an important cereal crop whose total global production was 881 million tons in 2016 [1]. Global warming is contributing to climate change, which is imposing an unprecedented level of heat stress (HS), and this change led to major losses in crop production worldwide [2]. HS disrupts cellular homeostasis due to the generation of an excessive amount of reactive oxygen species (ROS) [3]. This causes oxidative stress to occur, such as an overaccumulation of hydrogen peroxide (H_2_O_2_), single oxygen (^1^O_2_), and superoxide radicals (O_2_^−^) [4], which leads to the generation of an excess of malondialdehyde (MDA), a decrease in membrane flexibility, stability, and permeability, and the inhibition of protein membrane polymerization [5,6]. As a sessile organism, plants developed a diverse and complex antioxidant defense system to alleviate oxidative stress through detoxification of excess ROS [7]. This antioxidant defense system comprises ROS-scavenging enzymes, such as catalase (CAT), superoxide dismutase (SOD), peroxidase (POD), glutathione reductase (GR), and ascorbate peroxidase (APX), and non-enzymatic antioxidants, such as carotenoids, glutathione (GSH), and ascorbate (AsA) [3,8,9]. Moreover, plants developed a series of complex and dynamic strategies at multiple levels. At the transcription level, transcription regulators and ROS-related genes provide a timely response to these deleterious environmental effects [2]. Previous studies demonstrated that various transcription factors (TFs) positively regulate the responses against different abiotic stresses such as WRKY (binding a specific DNA sequence through WRKYGQK peptide) and MYB-genes [10,11,12,13,14,15]. From these, some specific genes such as TaWRKY26, TaWRKY39, and TaMYB80 were identified in a mitigation role against HS [12,16] in wheat. Genetic modification offers a beneficial approach to enhance the endogenous levels of osmo-protectants and antioxidants in crops. However, due to complication and the time-consuming nature of genetically modified crops, these genes have limited usage in applied agriculture practice. Therefore, alternative approaches could be used in applied agriculture practices to increase plant resistance in major crops.

Melatonin (MT) recently attracted the attention of researchers due to its role in protection against numerous environmental stresses. Several in recent reviews in this respect are very interesting [17,18,19,20,21,22,23,24]. This molecule is regarded as a potent antioxidant and cellular protective agent, even when compared with GSH and vitamin C [25]. The modulation of an antioxidant enzyme’s level and its activity in order to improve the detoxification of ROS (H_2_O_2_, O_2_^−^) is considered to be fundamental to enhance stress tolerance of plants [26,27]. In addition, MT is capable of inducing gene expression under abiotic and biotic stresses, germination, growth, development activities etc., [10,28,29,30,31,32,33,34,35,36,37,38]. With respect to the role of melatonin in heat tolerance, many studies reported that exogenous treatment with MT enhances tomato seedling tolerance to high temperatures by improving their antioxidant defense system [39,40,41,42]. Furthermore, in cucumber [43], kiwifruit [44], onion, leek [45], maize [46,47], and radish [48], a protective effect of MT against HS was described.

The current study in wheat seedlings aimed to elucidate (1) the role of MT in the regulation of the expression of stress-specific genes, (2) the influence of MT on chlorophyll content for the purpose of predicting photosynthetic efficiency, and (3) the role of MT in modulation of the antioxidant defense machinery for the purpose of understanding systemic stress-induced injuries.

## 2. Results

### 2.1. Melatonin Improved Growth Parameters in Wheat Seedlings under Heat Stress

The fresh weight (FW) and dry weight (DW) of shoots and roots from the seedlings significantly decreased under HS (42 °C) (Table S1 supplementary material). Conversely, exogenous treatment with MT mitigated the temperature-induced inhibition of growth components and facilitated better growth.

### 2.2. Melatonin Preserved the Integrity of the Cellular Membrane in Wheat Leaves under Heat Stress

**Figure 1:**
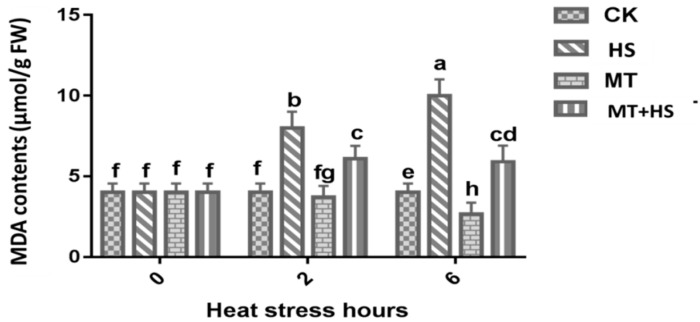
Effects of melatonin (MT; 100 μM) on malondialdehyde (MDA) content in leaves of wheat seedlings in the presence or absence of high temperature (42 °C). CK: control; HS: heat stress (42 °C); MT: melatonin (100 μM); MT + HS: melatonin (100 μM) + heat stress (42 °C). Data are means of three biological replicates (*n* = 3). Different letters indicate significant differences at *p* < 0.05 (ANOVA and Tukey HSD test); means ± SD.

The lipid peroxidation in plant membranes is usually determined by measuring the MDA content. The high-temperature treatment damaged the cellular membranes in the leaves of wheat seedlings after exposure to HS for up to 6 h. Exogenous application of MT (100 μM) was more effective at overcoming the severe impacts of HS-induced damage. A substantial decline in MDA content in MT + HS and MT leaves at 2 and 6 h was observed (Figure 1). An almost five-fold decrease in the MDA content was recorded at 6 h in comparison to CK (control).

### 2.3. Melatonin Controlled the ROS and Proline Content in Wheat Seedlings under Heat Stress

**Figure 2:**
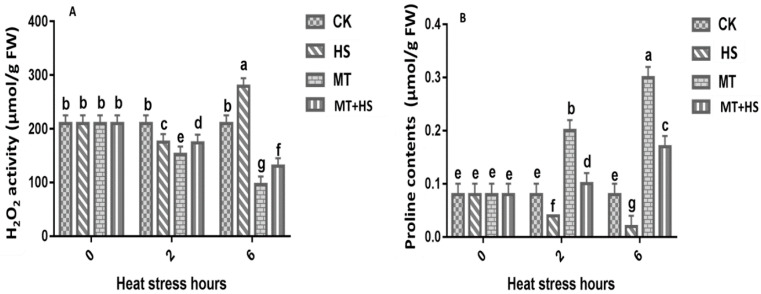
Effects of MT (100 μM) on (**A**) H_2_O_2_ and (**B**) proline content in leaves of wheat seedlings in the presence or absence of high temperature (42 °C). CK: control; HS: heat stress (42 °C); MT: melatonin; MT + HS: melatonin (100 μM) + heat stress (42 °C). Data are means of three biological replicates (*n* = 3). Different letters indicate significant differences at *p* < 0.05 (ANOVA and Tukey HSD test); means ± SD.

Plants produce H_2_O_2_ not only under normal conditions but also under stressful conditions. To determine whether MT alleviates heat-induced oxidative stress, we evaluated the trends of ROS accumulation in MT-pretreated leaves under HS at different time points by detecting H_2_O_2_ production (Figure 2A). At the initial stage (0 h), there was no difference in H_2_O_2_ production at all time points. However, compared with the control, a sharp increase in H_2_O_2_ production was observed in the leaf tissue at 6 h by HS. In contrast, the application of MT inhibited the generation of harmful H_2_O_2_ at 6 h, indicating that MT has a mitigating and protective effect. The analysis of the proline (Pro) content in different groups at different intervals is summarized in Figure 2B. Under HS conditions, the Pro content in MT-treated leaves was remarkably increased after 2 h in comparison to the corresponding control seedlings. After extending the heat stress until 6h, there was a significant decline in Pro content in HS treatment. In contrast, MT-treated leaves showed a higher increase in Pro content till 6 h.

### 2.4. Melatonin Increased the Antioxidant Enzyme Activity in Wheat Seedlings under Heat Stress

**Figure 3:**
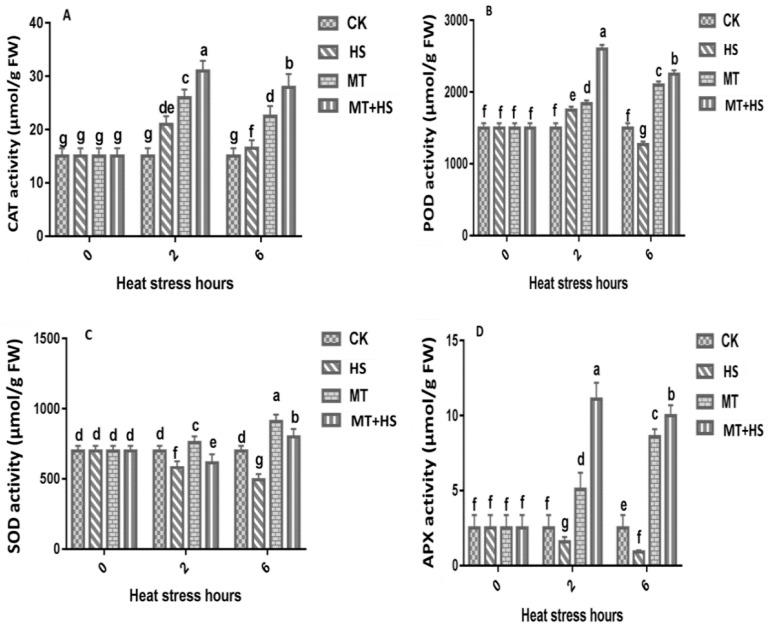
Effects of MT (100 μM) on antioxidant enzymes activity. (**A**) Catalase (CAT), (**B**) peroxidase (POD), (**C**) superoxide dismutase (SOD), and (**D**) ascorbate peroxidase (APX) in leaves of wheat seedlings in the presence or absence of high temperature (42 °C). CK: control; HS: heat stress (42 °C); MT: melatonin; MT + HS: melatonin (100 μM) + heat stress (42 °C). Data are means of three biological replicates (*n* = 3). Different letters indicate significant differences at *p* < 0.05 (ANOVA and Tukey HSD test); means ± SD.

The role of MT in the suppression of oxidative stress was investigated, and the activities of antioxidant enzymes at 0, 2, and 6 h were recorded (Figure 3). The MT pretreatment had a positive effect on the activities of CAT, POD, and APX when the plants were subjected to HS (42 °C). Compared with the control plants, HS increased the CAT activity by up to 20-fold. In the MT and MT + HS seedlings, the CAT activity was increased by 25-fold and 30-fold, respectively (Figure 3A). Similarly, the POD activity in seedlings treated with MT + HS and MT increased to 2700 µmol/g fresh weight (FW) and 1900 µmol/g FW, respectively, at 2 h (Figure 3B). A sharp decrease in POD activity was observed in leaves under HS at 6 h. The SOD activity decreased remarkably under HS and reached its lowest level of 500 µmol/g FW and 650 µmol/g FW at 6 h and 2 h, respectively, compared to the control and MT seedlings at 2 h and 6 h (Figure 3C). The MT + HS pretreated seedlings showed greater APX activity in the presence of HS, whereas the APX activity in the HS-treated seedlings was found to be reduced compared with that in the control (Figure 3D). A slight decrease in APX activity was observed at 6 h in MT-treated seedlings compared with the MT + HS-treated seedlings.

### 2.5. Glutathione Reductase (GR) Activity

**Figure 4:**
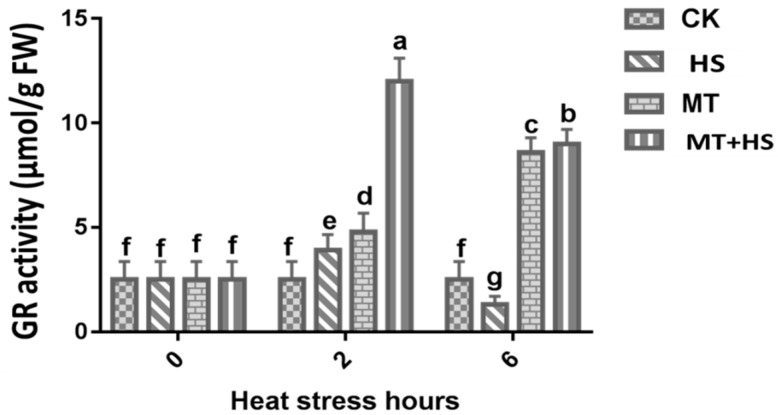
Effects of MT (100 μM) on glutathione reductase (GR) activity in leaves of wheat seedlings in the presence or absence of high temperature (42 °C). CK: control; HS: heat stress (42 °C); MT: melatonin (100 μM); MT+ HS: melatonin (100 μM) + HS (42 °C). Data are means of three biological replicates (*n* = 3). Different letters indicate significant differences at *p* < 0.05 (ANOVA and Tukey HSD test); means ± SD.

A remarkable increase in the GR activity was observed at 2 h, where it reached a maximum of 14.6 µmol/g FW. However, a decrease in the GR activity was observed at 6 h, where it reached a minimum of 10 µmol/g FW in the MT and MT + HS-treated leaves (Figure 4).

### 2.6. Exogenous Melatonin Induced the Endogenous Melatonin in Wheat Seedlings

**Figure 5:**
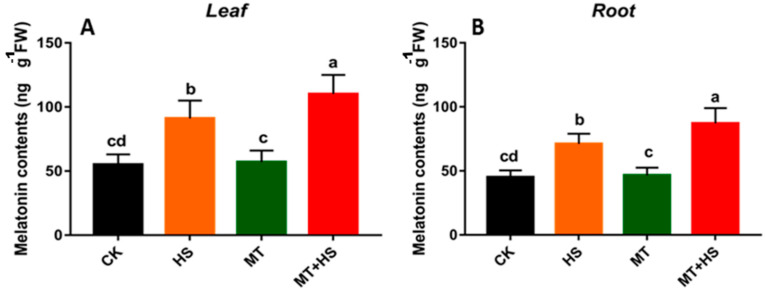
Evaluation of endogenous MT contents in (**A**) leaf and (**B**) root. CK: control; HS: heat stress (42 °C); MT: melatonin (100 μM); MT + HS: melatonin (100 μM) + HS (42 °C). The 10-day-old wheat seedlings were used to quantify the endogenous melatonin content. Different letters indicate significant differences at *p* < 0.05 (ANOVA and Tukey HSD test); means ± SD.

We investigated the effect of exogenous MT on the endogenous MT contents in wheat seedlings with and without stressor. The results indicated that the application of exogenous MT did not change the endogenous MT content in the absence of HS. In leaf tissue, significant changes were observed in the HS and MT + HS-treated plants where the endogenous MT contents rose to 95 and 115 ng·g^−1^ FW, respectively. Similarly, in root, the endogenous MT content respectively reached to its maximum of 71 and 87 ng·g^−1^ FW in HS and MT + HS-treated seedlings (Figure 5B). On the other hand, no significant differences were observed between control (CK) and MT-treated seedlings, with respect to endogenous MT content.

### 2.7. Exogenous Treatment with Melatonin Had a Protective Effect on Photosynthesis in Wheat Seedlings under Heat Stress

**Figure 6:**
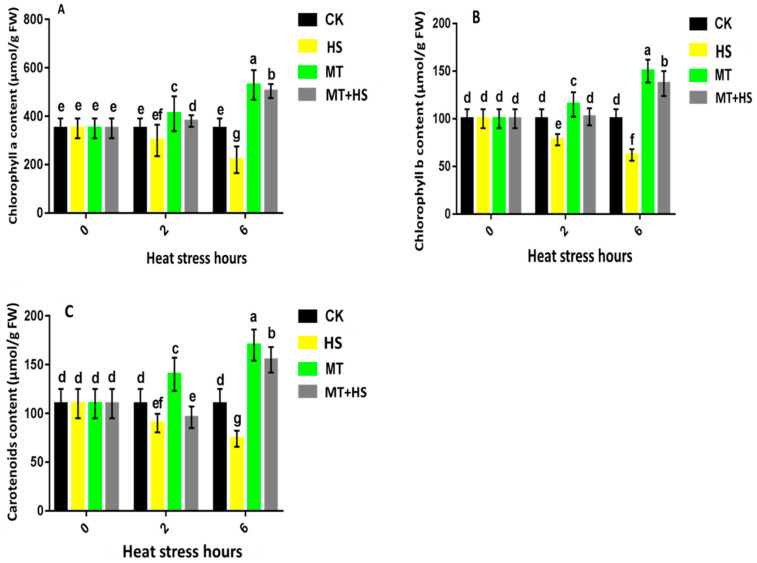
Effects of MT (100 μM) on chlorophyll contents. (**A**) Chlorophyll (Chl) a, (**B**) chlorophyll (Chl) b, and (**C**) carotenoid contents in leaves of wheat seedlings in the presence or absence of high temperature (42 °C). CK: control; HS: heat stress (42 °C); MT: melatonin (100 μM); MT + HS: melatonin (100 μM) + heat stress (42 °C). Data are means of three biological replicates (*n* = 3). Different letters indicate significant differences at *p* < 0.05 (ANOVA and Tukey HSD test); means ± SD.

A slight difference was observed between CK, MT, and MT + HS for chlorophyll a content at 2 h, where it reached its highest value of 410 µmol·g^−1^ FW (MT). However, a remarkable shift was observed at 6 h, where the chlorophyll a content reached its peak value of 600 µmol/g FW under MT and MT + HS (Figure 6A). Similarly, for chlorophyll b, the highest values of 155 and 150 µmol/g FW were recorded at 6 h under MT and MT + HS, respectively (Figure 6B). Regarding the carotenoid content, the lowest values of 90 and 75 µmol/g FW were recorded under HS at 2 and 6 h, respectively, in comparison to CK. At 6 h, the carotenoid content increased sharply under MT and MT + HS, where the value reached its peak of 170 and 150 µmol/g FW, respectively (Figure 6C).

### 2.8. Expression of the Antioxidant Enzyme Genes TaPOD, TaCAT, and TaSOD and Heat-Stress-Specific Genes

**Figure 7:**
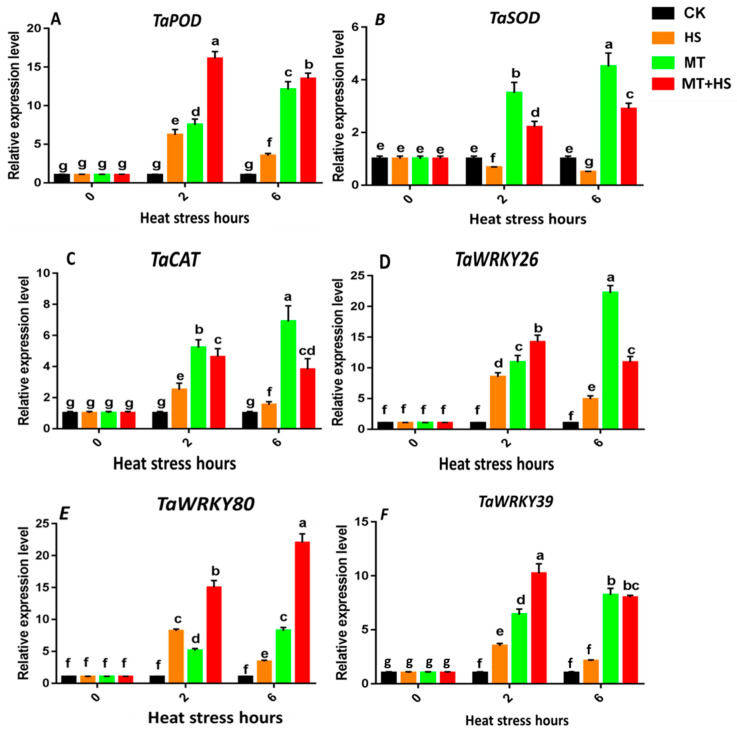
Effects of MT (100 μM) on antioxidant related genes. (**A**) *TaPOD*, (**B**) *TaSOD*, (**C**) *TaCAT*, and stress-specific genes (**D**) *TaWRKY26*, (**E**). *TaMYB80*, and (**F**); *TaWRKY39* in leaves of wheat seedlings in the presence or absence of high temperature (42 °C). CK: control; HS: heat stress (42 °C); MT: melatonin (100 μM); MT + HS: melatonin (100 μM) + heat stress (42 °C). Data are means of three biological replicates (*n* = 3). Different letters indicate significant differences at *p* < 0.05 (ANOVA and Tukey HSD test); means ± SD.

We observed a significant increase in the *TaPOD* expression level that peaked to 15-fold at 2 h in the MT + HS-treated leaves. However, a slight decrease was observed at 6 h, when the expression level dropped to 12-fold under MT and MT + HS (Figure 7A). A persistent increase in the *TaCAT* expression level was observed under MT; it rose to six-fold and 10-fold at 2 h and 6 h, respectively. Under HS, a down regulation of *TaCAT* expression was recorded at 2 and 6 h in comparison to that under MT; however, the expression level was restored to five-fold and four-fold at 2 h and 6 h, respectively (Figure 7C). The expression level of the *TaSOD* gene was also analyzed (Figure 7B). The *TaSOD* expression level was strongly increased under MT and MT + HS; it reached a maximum of four- and five-fold and two- and three-fold at 2 h and 6 h, respectively.

Stress-specific genes from the WRKY and MYB TF families were selected because of their involvement in increasing tolerance to heat stress (Figure 7D–F). The *TaMYB80* expression showed a significant increase in the MT and MT + HS-treated plants. The dominant expression trend was observed under MT + HS, in which the highest expression of 24-fold was recorded at 6 h followed by an expression level of 15-fold at 2 h. The *TaWRKY26* expression reached a peak of 25-fold at 6 h under the MT treatment, whereas the expression was down regulated under the HS treatment at both 2 and 6 h. Regarding *TaWRKY39*, the transcriptional level was significantly increased under both MT + HS and MT treatments, in which the highest values of 10-fold and eight-fold were recorded at 2 and 6 h, respectively.

## 3. Discussion

**Figure 8:**
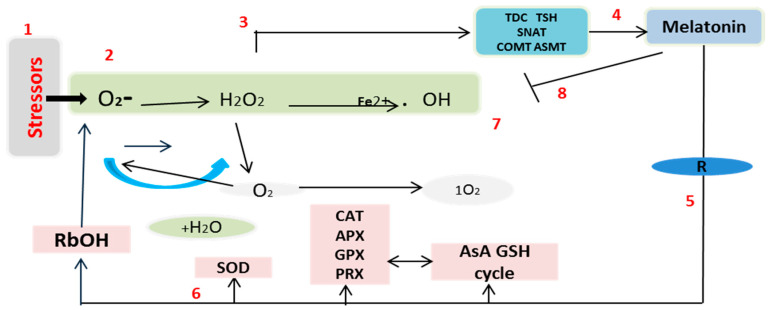
(**1**) The biotic and abiotic stresses triggered the production of ROS. (**2**) The increase of ROS level generates oxidative stress. (**3**) ROS (especially O_2_^−^, H_2_O_2_), are capable of inducing the expression of melatonin biosynthesis genes (TDC, T5H, SNAT, and COMT, ASMT). (**4**) An increase in melatonin levels occurs as a result of the biosynthesis of endogenous melatonin. This response can be stimulated or reinforced by exogenous melatonin. (**5**) Melatonin, through interaction with its receptor (*R = CAND2/PMTR1*), induces the expression of several enzymes such as (**6**) RbOH and SOD, increasing O_2_^−^ and H_2_O_2_ levels, among others (**7**) As a result, ROS levels are controlled by Antioxidative enzymes (CAT, APX, GPX, PRX, AsA GSH) and (**8**) also regulated by the direst action of melatonin (and its by-products) through their scavenging action.

Wheat is one of the world’s major cereal crops. However, its production is at the mercy of numerous environmental stresses [16]. MT is a pleiotropic molecule well known for the multiple roles it plays in plants, including germination, growth and root architecture, photosynthesis, water economy, and primary and secondary metabolisms [17,18,19,24,28,49,50]. Moreover, MT provides defense against biotic and abiotic stresses, increasing plant tolerance. Cellular oxidative damage is a marker of HS, and the generation of MDA and ROS results from this oxidative stress. This causes an increase in the permeability of plant membranes and damages the integrity of the membrane’s structure. Importantly, H_2_O_2_ works like a double-edged sword. At low concentrations, it is beneficial to plants, acting as a messenger molecule [25,51], but it has a detrimental effect at high concentrations in the cell [52]. Under prolonged HS, the wheat leaves produced an excessive amount of H_2_O_2_, which hinders plant growth and development processes. The treatment with MT of wheat seedlings under high-temperature HS resulted in a sharp decrease in H_2_O_2_ (Figure 2A). These results are consistent with previous findings in kiwifruit [44] and cucumber [43], among others [46,47,48]. The results can be partly attributed to the fact that MT is a free radical scavenger and a broad-spectrum antioxidant, and it can eradicate ROS involved in cellular disfunction, as displayed in Figure 8. MDA also represents an important stress indicator that forms as a result of ROS-induced biological membrane damage through an auto-oxidative chain reaction [53]. As reported above, as the time of exposure to HS increased from 2 to 6 h, there was a sharp increase in the MDA content in the wheat seedlings that could potentially have damaged the plasma membrane’s integrity (Figure 1). Furthermore, treatment with MT decreased the MDA content; this result is consistent with previous findings on tomato [54], Bermuda grass [55], and kiwifruit [44] under different abiotic stresses [17,18,49]. Taken together, these results indicate that MT reduces heat-induced oxidative damage by balancing ROS during exposure to high temperature, and it may be able to repair the disrupted cellular membrane. The model shown in Figure 8 is in accordance with the most recent published integrative system in which MT acts as a main regulator, controlling the redox network in plants, regulating ROS level, antioxidative enzymes and metabolites, and its own biosynthesis, upregulating the melatonin biosynthesis enzymes [17,27].

A relevant indicator of a plant’s resistance to abiotic stress is the changes in the chlorophyll levels due to degradation. The present study showed that MT plays a role in the detoxification of ROS, as well as in the maintenance of the chlorophyll content (Figure 6). Photosynthetic pigment biosynthesis, which encodes chlorophyll a and b and carotenoids, was found to be affected under all of the stress phases; however, exogenous application of MT was found to restore the chlorophyll content in comparison to the control. Previous studies also reported that the effects of short- and long-term HS can be mitigated by the exogenous application of MT [56,57]. Furthermore, MT clearly retards dark-induced senescence in leaves, protecting photosynthesis pigments [33]. These evidences indicate that MT is a positive plant regulator in harsh environments as it enhances the capacity of plants to tolerate HS [19].

MT is surprisingly effective in a remarkably large range of circumstances [17,18,19], including the improvement of enzymatic antioxidants, such as POD, SOD, CAT, GR, and APX antioxidant scavenging activity to control cellular redox homeostasis [58]. Therefore, modulating the antioxidant defense system through the detoxification of excess ROS may help to mitigate the deleterious effects of abiotic stressors, including HS [27]. The antioxidant defense-related enzymes and non-enzymes showed a decrease in their activity when exposed to an extended period of HS (Figure 3A–C). However, POD, SOD, and CAT activities in wheat seedlings treated with MT showed a remarkable boost compared with the control. The first line of defense for ROS detoxification in plants is based on SOD activity, which converts ROS into H_2_O and O_2_ by eliminating O_2_^−^ [59]. In addition, POD and CAT were also found to aggressively contribute to H_2_O_2_ scavenging through converting it into O_2_ and H_2_O [60], which signify the role these antioxidant enzymes play in H_2_O_2_ detoxification. These results are consistent with previous findings on MT-treated kiwifruit [44], tea [61], and wheat [62], in the presence of different stressors. The AsA–GSH cycle represents an important way to eliminate free radicals in plants. For this reason, MT-induced production of GSH and AsA was linked to a low level of H_2_O_2_ production in abiotic stress [63]. The AsA and GSH in the cells were oxidized [64]. The APX activity increases significantly upon exposure to biotic or abiotic stresses [65,66]. In the current study, we used APX and GR as indicators to evaluate the GSH cycle in different groups of wheat seedlings. We observed that the APX and GR content decreased under the HS treatment and when increasing the duration of exposure to HS. On the other hand, in the MT-pretreated leaves, we observed a significant increase in APX activity after prolonged exposure to HS (6 h) (Figure 3D). This indicates that APX can detoxify H_2_O_2_ into H_2_O and produce MDA. Our findings suggest that, in the MT-treated plants, the APX and GR activities increased in order to improve GSH biosynthesis to maintain a critical balance between ROS accumulation and the ROS-scavenging mechanism. Pro is an amino acid involved in the osmoregulation process, and it can also be regarded as an antioxidant used to counter the damaging effects of different ROS. During exposure to stress, Pro accumulation in plants is associated with increased biosynthesis and reduced degradation [67]. The production of Pro was found to be similar in all groups of wheat seedlings after 2 h. A further increase in the duration of exposure to HS produced a significant difference between MT-treated and untreated seedlings (Figure 2B). This result demonstrates the strong and effective co-regulation by Pro and MT that imparted partial heat tolerance to the wheat seedlings by reducing the number of cellular injuries and protecting some of the vital enzymes related to antioxidant machinery, which enhanced the plants’ ability to deal with heat-induced damage [68]. Previous experimental results in chickpea support this finding [69]. The direct uptake of exogenous MT by roots was reported in previous studies [70]. The endogenous level of MT increased when the plants were exposed to environmental stresses [17]. The induction in endogenous MT level is crucial in triggering the plant response to these adverse conditions [17]. Subsequently in our study, the level of endogenous MT increased significantly under HS and MT + HS (Figure 5). As commented above, different stressors clearly activate MT biosynthesis upregulating MT biosynthesis enzymes. Data in Figure 5 are in accordance with this endogenous MT biosynthesis increase. Therefore, the applications of MT help the plants to maintain an optimal level of endogenous MT in leaves and roots, counteracting the negative effect of stressors [19,27,34].

ROS induce oxidative stress at high concentration and act as a signaling molecule at low concentration. *TaSOD*, *TaPOD*, and *TaCAT* are defense-related genes involved in detoxification of excessive ROS. The antioxidant-related genes *TaCAT*, *TaPOD*, and *TaSOD* displayed a high transcriptional level under MT and HS groups at 0 h to 2 h. However, a sharp decrease was observed with increasing heat stress exposure time until 6 h. This was consistent with the H_2_O_2_ production under the HS treatment and in the control leaves. This indicates that the exogenous MT treatment had a beneficial effect on the tolerance to oxidative stress by removing the excessive H_2_O_2_.

The WRKY and MYB transcription factors were shown to have important roles in the regulation of complex and dynamic characteristics under different conditions, including abiotic stress. However, relatively little information is available on heat-responsive genes in wheat. In the highly developed dynamic network of defense mechanisms, the stimulation of stress-responsive genes plays a main role [71]. We analyzed the transcription of the stress-responsive genes *TaWRKY39* and *TaWRKY26* from the WRKY gene family under various treatments (Figure 7). There was a significant increase in the expression level of *TaWRKY39 and TaWRKY26* under MT and MT + HS [72,73]. MT works as a growth regulator under HS conditions, which was reported in previous studies. The induction of *TaWRKY39* and *TaWRKY26* transcription under various growth regulators, such as salicylic acid (SA), jasmonate (JA) [74], and ethylene [75], was previously analyzed. Additionally, an effective connection between MT and different plant hormones was studied [21,38]. Together, these findings reveal that MT positively regulates both genes and facilitates the response to HS. To date, *Arabidopsis thaliana* is reported to have 204 *MYB*s, whereas 218 and 244 *MYBs* were identified in rice (*Oryza sativa*) [75] and soybean (*Glycine max*) [76], respectively. Accumulating evidence indicates that MYBs are involved in the strengthening of stress signaling pathways. The expression profile of different MYB genes was reported in multiple stress treatment studies. The exogenous application of MT significantly enhanced the relative *TaMYB80* gene expression level in MT + HS-treated plants after an extended period of exposure to HS at 6 h (Figure 7E), as also shown by [77]. These results confirm that the overexpression of *TaMYB80* in leaf tissue is further strengthened upon exogenous application of MT.

## 4. Materials and Methods

### 4.1. Plant Material and Experimental Details

We used Shannong 33 (*Triticum aestivum* L.) plants. The wheat seeds were pretreated with 1% sodium hypochlorite for 10 min. Then, seeds were washed with distilled water 3–5 times, placed on petri plates with filter paper, and allowed to germinate for three days. For hydroponic culture, wheat seedlings were grown for seven days in 1/4 Hoagland solutions. The seedlings were divided into two sub-groups for challenge under different treatments. In the first sub-group, we sprayed 80 mL of 100 μM MT on each of the wheat seedling leaves each day and for seven days. In the second sub-group, the same volume of water was sprayed on each of the wheat seedling leaves. After one week, half of the water-treated seedlings and half of the MT-treated seedlings were removed and exposed to high temperature (42 °C). This protocol resulted in four groups of plants: (1) a control group (CK); (2) an MT (100 μM) treatment group; (3) an HS (42 °C) treatment group; (4) an HS (42 °C) and MT (100 μM) treatment group. The MT concentration was selected based on a previous experiment [39,78]. A stock solution of standard MT (Sigma-Aldrich, St. Louis, MO, USA) was prepared by dissolving MT in ddH_2_O (0.01% v/v). Tween-20 was used as a surfactant. All experiments were conducted in a growth chamber at Northwest Agriculture and Forestry University, where environmental conditions were maintained at 25/20 °C (day/night) with a relative humidity of 50% ± 5%, a light intensity of 450 µmoL·m^−2^·s^−1^, and a 12/12-h (light/dark) photoperiod. There were at least three biological replicates per treatment.

The plants in the CK group were maintained at 25 °C throughout the experiment. The plants in the HS (42 °C) group were moved to growth cabinet (Percival Scientific, Inc, Perry, IA, USA) with settings as above but exposed to 42 °C to stress the plants, over 2 h, and then sustained at 42 °C to the 6-h time point. All the control samples were collected at 0 h, which was also used as the starting point before extending the stress periods to 2 and 6 h, respectively [79]. All collected samples were immediately frozen in liquid nitrogen and then stored at −80 °C.

### 4.2. Measurement of Growth Indicators

To assess combined effect of MT and HS in wheat seedlings, we measured different growth indicators such as fresh and dry weight of shoots and roots. For dry weight determination, plants were oven-dried at 65 °C for overnight.

### 4.3. Calculation of Malondialdehyde (MDA), Proline (Pro), Hydrogen Peroxide (H_2_O_2_), Chlorophyll (Chl), and Carotenoid Content

The thiobarbituric acid method was used to measure the MDA content [80]. Briefly, 0.3 g of wheat leaves were crushed by using 5 mL of cold 5% (*w*/*v*) trichloroacetic acid solution (TCA) and centrifuged at 10,000× *g* for 10 min at 4 °C. Then, 2 mL of supernatant and 0.67% (*w*/*v*) thiobarbituric acid (TBA) was added, and the solution was sealed, mixed, and heated for 10 min in a bath of boiling water. The absorbance of the supernatant was measured at 450, 532, and 600 nm following the bath and then cooling. The H_2_O_2_ concentration was determined based on the method described in Reference [81]. Briefly, 0.3 g of wheat leaves was ground to a homogenate with 5 mL of cold acetone and centrifuged for 10 min at 4 °C and 10,000× *g*. Then, 1 mL of supernatant was added to 0.1 mL of 5% (*w*/*v*) titanium sulfate and 0.2 mL of concentrated ammonia, and the solution was mixed. Then, the mixture was centrifuged for 10 min at 4 °C. Subsequently, the precipitate was removed and washed 3–5 times with acetone until the plant’s pigment was removed. Finally, the precipitate was dissolved in 5 mL of 2 M concentrated sulfuric acid, and the volume of distilled water was fixed to 10 mL. The absorbance was measured at 415 nm. The H_2_O_2_ content was based on a standard curve generated with known H_2_O_2_ concentrations. The chlorophyll concentration was measured as described by [82]. The chlorophyll and carotenoid contents were measured by following the method of [82]. Briefly, 0.1 g of fresh leaf sample was sliced and placed in glass test tubes. Then, 10 mL of 80% acetone was added to each tube, and the tubes were placed in the dark until the leaf samples were completely discolored. The extract was then centrifuged, and the absorbance of the supernatant was measured at 645, 663, and 440 nm in order to determine the chlorophyll a and b and carotenoid content using an ultraviolet (UV) spectrophotometer (trademark, model, country). Leaf segments (0.5 g) were taken from each replicate in order to measure the Pro content following [83]; the quantity was measured at 528 nm on a spectrophotometer using a standard curve [84].

### 4.4. Antioxidative Enzyme Analysis

The CAT, POD, and SOD activities were measured by following the method of [85]. Briefly, to extract a crude enzyme solution, 0.3 g of leaf sample was ground to a homogenate in 8 mL of cold 50 mM PBS (pH 7.8) containing 1% (*w*/*v*) polyvinylpyrrolidone (PVP), 2 mM dithiothreitol (DTT), and 0.1 mM ethylene diaminetetraacetic acid (EDTA). The homogenates were then centrifuged for 10 min at 4 °C and 10,000× *g*, and the supernatant was saved for further analysis of enzyme activity. The POD activity was measured by using guaiacol as a substrate. The reaction mixture contained 50 mM PBS (pH 5.5), 30% (*v*/*v*) H_2_O_2_, guaiacol, and the enzyme extract. The variation in absorbance at 470 nm was recorded and the result was expressed as µmol/g (a decrease in absorbance of 0.01 per min is 1 U at 470 nm). The SOD activity was measured based on the photochemical reduction of nitro blue tetrazolium (NBT) and monitoring the absorbance at 560 nm. The result was expressed as µmol/g (the absorbance of 1 g of leaf sample in the reaction mixture at 470 nm is reduced by 0.01 per minute in 1 U). The CAT activity was measured by monitoring the decrease at 240 nm and the result was expressed as µmol/g (the absorbance of 1 g of leaf sample in the reaction mixture at 470 nm is reduced by 0.01 per minute in 1 U). The reaction mixture consisted of 100 mM H_2_O_2_, 200 mM PBS (pH 7.8), and the enzyme extract.

The APX activity was measured by following the method of [86]. Briefly, 0.5 g of wheat leaves were ground in a chilled mortar with 4% (*w*/*v*) polyvinylpolypyrrolidone, then homogenized with 8 mL of 50 mM phosphate buffer (pH 7.5) containing 1 mM EDTA–Na_2_ and 0.3% Triton X-100. The reduction in absorbance at 290 nm was measured as H_2_O_2_ was oxidized. The GR activity was assayed by using the method described in [87]. Briefly, we mixed 100 µL of 100 mM tricine (pH 7.6), 20 µL of 3.24 mM NADPH, 50 µL of extract, and 20 µL of 16 mM oxidized glutathione (GSSG). The oxidation of NADPH was measured at 340 nm (ε = 6.22 mM^−1^·cm^−1^) in a microtiter plate reader using equipment.

### 4.5. RNA Preparation and Gene Expression Analysis

A 0.1-g sample of wheat (*Triticum aestivum* L.) leaves was used to extract total RNA by using a Total RNA kit (TIAGEN, Beijing, China) following the manufacturer’s instructions. For the complementary DNA (cDNA) synthesis, 2 mg of total RNA was reverse-transcribed using a PrimeScript TM II First-Strand cDNA synthesis Kit (TakaRa Dalian, China). The quantitative real-time PCR (qRT-PCR) analysis was performed with a Light Cycle 480 II system (Roche, Basel, Switzerland) using an SYNR Premix Ex TaqTM kit (TakaRa Dalian, China). The *UBI-eq* gene was used as an internal control. The selected stress-responsive genes were from wheat *(Triticum aestivum* L.). The primer sequences used for the PCR are given in Table S1 (Supplementary Materials). Three replicates were made for three separate RNA extracts from the three samples.

### 4.6. Quantification of Endogenous Melatonin

The 10-day-old wheat seedlings were sprayed with MT. The roots and leaves were collected to examine the endogenous MT contents by following the method of Byeon and Back [88]. HPLC (Shimadzu, Kyoto, Japan) was used for analyzing the endogenous MT contents in wheat seedlings. Dried and frozen tissues of 0.5 g were ground and then 5 mL of chloroform was added. The mixture was centrifuged at 10,000× *g* for 10 min with temperature set at 4 °C. The samples were left in room temperature in order to let the chloroform evaporate. The parameters 0–27 min, 42–50% methanol and 27–45 min, 50% methanol at a flow rate of 0.15 mL·min^−1^ were used to prepare the sample for determining endogenous MT contents. The fluorescence detector was programmed with an excitation value of 280 nm and 348 nm for emission. To obtain the standard curve, we used standard melatonin. Three biological replicates were used for all the samples.

### 4.7. Statistical Analysis

All of the data were statistically analyzed with SPSS software (version 25.0, SPSS Inc., USA). One-way analysis of variance was performed, and statistically significant differences among the treatments were determined using Tukey’s honest significant differences test at *p* < 0.05. The data were plotted using GraphPad Prism 7.0 (GraphPad Software, Inc., LA Jolla, CA, USA).

## 5. Conclusions

The probable underlying mechanisms via which exogenous application of MT mitigates the induction of oxidative damage and modulates the antioxidant defense system in wheat seedlings are proposed in the schematic diagram shown in Figure 8. The exogenous application of MT enhances the thermal tolerance of wheat seedlings by reducing the formation of ROS, enhancing the antioxidant enzyme systems, activating the AsA–GSH cycle, increasing the presence of osmolytes (Pro), and maintaining equilibrium between ROS production and antioxidant scavenging activities through upregulation of their related genes. Hence, we conclude that HS-induced damage was minimized by MT, which coordinates with the antioxidant defense system by increasing the biosynthesis of antioxidant enzymes and helping to detoxify excess ROS. Furthermore, HS induces endogenous MT biosynthesis, preparing plants for stress. These findings provide novel insights into the cross-talk that occurs between MT and specific stress-responsive genes in wheat seedlings to inhibit thermal stress. However, the proposed mechanisms via which MT and the antioxidant defense system protect against abiotic stress must be further characterized in additional plants.

## Supplementary Materials

The following are available online at https://www.mdpi.com/2223-7747/9/7/809/s1, Figure S1: title, Table S1: Effects of melatonin on the morphology of heat stress exposed wheat seedlings, Table S2 Primer sequences used in this study.

## Abbreviation

**Abbreviation**	**Full Name**
MT	Melatonin
HS	Heat stress
Pro	Proline
SOD	Superoxide dismutase
POD	Peroxidase
CAT	Catalase
MDA	Malondialdehyde
APX	Ascorbate peroxidase
ROS	Reactive oxygen species
H_2_O_2_	Hydrogen peroxide
GR	Glutathione reductase
FW	Fresh weight
DW	Dry weight
AsA–GSH	Ascorbate–glutathione
NADPH	Nicotinamide adenin dinucleotide phosphate
SNAT	Serotonin N-acetyl transferase
TDC	Tryptophan decarboxylase
GPX	Glutathion peroxidase
PRX	Peroxirredoxin
ASMT	Acetylserotonin methyltransferase
RbOH	Respiratory burst oxidase homolog
COMT	Caffeoyl-O-methyl transferase
T5H	Tryptamine 5-hydroxylase
SNAT	Serotonin N-acetyl transferase
