# Supplementary material for: Melatonin Suppressed the Heat Stress-Induced Damage in Wheat Seedlings by Modulating the Antioxidant Machinery
Source: Plants (Basel). 2020 Jun 28;9(7):809. doi: 10.3390/plants9070809 (PMC7412093; doi:10.3390/plants9070809)
Supplement: Supplementary file 1 [file plants-09-00809-s001.pdf]

Table S1 Effects of melatonin on the morphology of heat stress exposed wheat seedlings.

Treatments	Shoot fresh weight (g plant^{-1})	Shoot dry weight (g plant^{-1})	Root fresh weight (g plant^{-1})	Root dry weight (g plant^{-1})
CK	0.87±0.025a	0.12±0.03a	0.65±0.016b	0.08±0.01ab
HS	0.61±0.18b	0.08±0.02c	0.55±0.15a	0.06±0.002a
MT	0.89±0.15b	0.15±0.06a	0.75±0.13b	0.09±0.02a
MT+HS	0.64±0.16a	0.14±0.04b	0.70±0.21a	0.08±0.02a

Data represent as a mean of standard deviation (SD) of three replications. Different letters indicate significant differences according to Tukey's HSD test at $P \leq 0.05$. Ck-Control; HS: Heat stress (42°C); MT-melatonin (100 μM); MT + H**S**: melatonin (100 μM) + heat stress (42 °C)

Table S2. Primer sequences used in this study

**Gene Name**	**Sequence**
TaMYB80<br>*TaMYB80*	F-5'GCCCAATCCGTCTCTACTCTA<br>R-5'AGGTGGAACCCGTACTTGTAT
TaWRKY26<br>*TaWRKY26*	F-5'ATGTCCTCCTCCACGGGGA<br>R-5'CTAGCAGAGGAGCGACTCGACG
TaWRKY39<br>*TaWRKY39*	F-5'TTTGAGCTCAACAATCGTGATTCGTGAT<br>R5'TTTCCATGGTTTTTCTTCTACCCAAGTTTCAGA
TaSOD	F-5'-TCCTTTGACTGGCCCTAATG-3'<br>R-5'-CTTCCACCAGCATTTCCAGT-3'
TaPOD	*F-5*'AGCACACAAGGAGAGAGGAG<br>*R-5*'AAGAGGCACGCGGTATCG
TaCAT	R: GTGCCTTGCCGATGGTGT<br>F: TGCCTGTGTTTTTTATCCGAGA
